# Seasonal dynamics and co‐occurrence patterns of honey bee pathogens revealed by high‐throughput RT‐qPCR analysis

**DOI:** 10.1002/ece3.5544

**Published:** 2019-08-14

**Authors:** Paul D'Alvise, Victoria Seeburger, Katharina Gihring, Mattias Kieboom, Martin Hasselmann

**Affiliations:** ^1^ Department of Livestock Population Genomics, Institute of Animal Science University of Hohenheim Stuttgart Germany; ^2^ Apicultural State Institute University of Hohenheim Stuttgart Germany; ^3^ Process, Energy & Environmental Technology Station, Faculty of Engineering and the Built Environment University of Johannesburg Doornfontein Campus Doornfontein South Africa; ^4^ Environmental Research Institute Wageningen University Wageningen The Netherlands

**Keywords:** black queen cell virus, honey bee, *Nosema ceranae*, pathogens

## Abstract

The health of the honey bee *Apis mellifera* is challenged by introduced parasites that interact with its inherent pathogens and cause elevated rates of colony losses. To elucidate co‐occurrence, population dynamics, and synergistic interactions of honey bee pathogens, we established an array of diagnostic assays for a high‐throughput qPCR platform. Assuming that interaction of pathogens requires co‐occurrence within the same individual, single worker bees were analyzed instead of collective samples. Eleven viruses, four parasites, and three pathogenic bacteria were quantified in more than one thousand single bees sampled from sixteen disease‐free apiaries in Southwest Germany. The most abundant viruses were black queen cell virus (84%), Lake Sinai virus 1 (42%), and deformed wing virus B (35%). Forager bees from asymptomatic colonies were infected with two different viruses in average, and simultaneous infection with four to six viruses was common (14%). Also, the intestinal parasites *Nosema ceranae* (96%) and *Crithidia mellificae/Lotmaria passim* (52%) occurred very frequently. These results indicate that low‐level infections in honey bees are more common than previously assumed. All viruses showed seasonal variation, while *N. ceranae* did not. The foulbrood bacteria *Paenibacillus larvae* and *Melissococcus plutonius* were regionally distributed. Spearman's correlations and multiple regression analysis indicated possible synergistic interactions between the common pathogens, particularly for black queen cell virus. Beyond its suitability for further studies on honeybees, this targeted approach may be, due to its precision, capacity, and flexibility, a viable alternative to more expensive, sequencing‐based approaches in nonmodel systems.

## Graphical abstract

A comprehensive set of qPCR assays was established for the Biomark system (Fluidigm), which allows parallel quantification of all relevant honey bee pathogens. The method was applied on single‐bee samples from asymptomatic colonies in Southern Germany, and prevalences, population dynamics, and co‐occurrence patterns of the pathogens were analyzed.

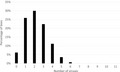

## 1 INTRODUCTION

The Western honey bee *Apis mellifera* is an important pollinator worldwide. Most honey bee colonies are managed by beekeepers, and they have been domesticated and bred toward gentleness and honey yield. This artificial selection may have compromised their inherent resistance against pathogens and parasites. Owing to their eusocial lifestyle with permanent colonies comprised of thousands of individuals in close contact, honey bees are prone to infections, and there is a considerable number of pathogens and parasites that may afflict them. There is a wide range of honey bee pathogens; more than twenty viruses, five or more pathogenic bacteria, four pathogenic/parasitic fungi, four parasitic protozoans, and three parasitic mites have been described (Bailey & Ball, 1991; Evans & Schwarz, 2011). During the recent years, many previously undescribed viruses were found in honey bees by untargeted RNA sequencing; however, it is unclear whether all these bee‐associated viruses actually infect honey bees (Grozinger & Flenniken, 2019; McMenamin & Flenniken, 2018; Remnant et al., 2017). Due to new introductions and international exchange of bees, parasites from the Eastern honey bee *Apis cerana*, that is, the mite *Varroa destructor* and the microsporidian *Nosema ceranae*, have been introduced to the Western honey bee and spread globally.

Since *V. destructor* made the host shift from *A. cerana* to *A. mellifera*, beekeepers in temperate zones have to deal with substantial colony losses, which occur mainly during winter. Besides high *Varroa* infestation, virus infections were identified as a major risk factor for winter losses (Genersch et al., 2010; Highfield et al., 2009). Infections with deformed wing virus (DWV) are associated with high *Varroa* infestation, since the mite acts as a vector for the virus, which is able to propagate inside it (Bowen‐Walker, Martin, & Gunn, 1999; Rosenkranz, Aumeier, & Ziegelmann, 2010; Wilfert et al., 2016). Yet, besides the known DWV‐mite interaction, the factors leading to disease outbreaks and colony losses are still poorly understood. Most bee scientists agree that colony losses cannot be attributed to one single factor, but to the interaction of different biotic and abiotic stressors (LeConte, Ellis, & Ritter, 2010). Interactions among viruses or other pathogens and parasites may facilitate excessive virus proliferation and development of disease. A recent study demonstrated that after experimental infection with different mixed virus inocula, virus proliferation was strongly influenced by what other viruses were present in the mixed inoculum (Carrillo‐Tripp et al., 2016). The authors concluded that complex virus‐virus interactions, such as competition for cellular resources or modulation of host defense systems, likely affect infection dynamics.

The goals of this study were to identify typical co‐occurrence patterns among pathogens and parasites, and to analyze their seasonal population dynamics in healthy colonies, serving the overall objective of identifying synergistic interactions between pathogens and/or parasites that may be the cause of increased pathogen proliferation and subsequent colony death. Taking advantage of improved molecular methods and automation, we have established a comprehensive set of assays for parallel quantification of pathogens and parasites. Considering the individual as most immediate level of pathogen/parasite interactions, we conduct the analyses in individual bees. This is in contrast to many preceding studies that have mostly analyzed collective samples consisting of multiple individuals, which does not provide information about the distribution and colocalization of pathogens and parasites within the individual bees. Therefore a single‐bee approach with many replicates was previously recommended, but considered to be not feasible due to technical and financial restrictions (Gauthier et al., 2007).

## 2 MATERIAL AND METHODS

### 2.1 Bee colonies

Fifteen colonies in five nonmigratory apiaries were sampled regularly throughout one year, from 2016 to 2017. Only samples from these regularly sampled colonies were used for assessing seasonal occurrence patterns. Forty‐two additional colonies in fourteen apiaries within the same area were sampled 1–4 times, mostly during the warm season. These additional samples were added to the sample pool for assessing prevalence, abundance, and co‐occurrence of pathogens and parasites. The apiaries are located within a radius of 100 km around Stuttgart (SW‐Germany) in cultural landscape with land use structured at small scales (forests, orchards, meadows, agricultural and residential areas) that, in general terms, offers sufficient and diverse forage. The minimal distance between the regularly sampled apiaries was 12 km. The apiaries were managed by different commercial and hobbyist beekeepers and were treated against *Varroa* mites exclusively by evaporating 60% formic acid in late summer and trickle‐application of 3.5% oxalic acid in sucrose solution during the brood‐free period in December. No visible disease symptoms were noted in the sampled colonies and none of the long‐term monitored colonies died during the study period.

### 2.2 Sampling

During the warm season, forager bees were sampled with transparent plastic bags that were held in front of the hive entrance until 15–20 bees had flown into the bag. Samples of winter bees were retrieved from inside the hive. The bee samples were immediately frozen in water ice covered with frozen thermal packs (−15 to −5°C) and transferred to −80°C within 6 hr, where they were stored until RNA extraction. For each time point, 18 bees per apiary were analyzed, from three colonies per apiary six bees were analyzed from each colony.

### 2.3 Extraction

RNA was extracted using a TRIzol protocol. Complete single bees were placed in a 2‐ml lysis tube with five 0.8‐mm steel beads, roughly 50 µl 0.1‐mm glass/zirconia beads and 0.5 ml TRIzol (Invitrogen). The bees were homogenized on a FastPrep24 (MP Bio) at 5.5 m/s for 50 s. After 5 min incubation at room temperature (RT), 100 µl chloroform were added and the contents were mixed by vigorous shaking, followed by another 5 min incubation at RT. The two phases were separated by 15 min centrifugation at 12,000 *g* and 4°C. 200 µl of the aqueous phase was transferred to a new tube with 250 µl isopropanol and mixed by repeated inverting. After another 10 min incubation at RT and 10 min centrifugation (12,000 *g*, 4°C), the supernatant was removed, the pellet was washed with 80% ethanol, dried for 5 min at RT, and redissolved in 50 µl nuclease‐free water. RNA concentrations were determined on a NanoDrop spectrophotometer (Thermo Fisher) and, for reference, on a Qubit fluorometer (Thermo Fisher). The resulting RNA concentrations ranged between 700 and 2,500 ng/µl (NanoDrop), which corresponded to about 200–900 ng/µl (Qubit). NanoDrop concentration measurements were used for calculations of dilution factors and as reference for pathogen concentrations.

### 2.4 Assay design

Assays for pathogens, parasites and control genes were either adopted from previous studies or newly designed to fit the reference sequences available at the database of the National Center for Biotechnology Information (NCBI) (Table 1). If more than one reference sequence was available, the reference sequences were aligned in CLC Main Workbench 7.4.1 (Qiagen Bioinformatics, Aarhus, Denmark) to identify conserved sequences as primer sites. Although particular care was taken to fit the assays to any known variant of the pathogens, unknown variation may lead to false‐negative findings and the true amount of pathogens may be underestimated. Every assay was tested by endpoint PCR, which was conducted with a final primer concentration of 0.2 µM according to the manufacturer's recommendations (DreamTaq Green, Thermo Fisher Scientific, PCR cycle: 2 min 95°C; 35 cycles 20 s 95°C, 30 s 60°C; 1 min 60°C, hold 4°). If available, a sample of bees that showed clinical symptoms of the respective pathogen was used as positive control. Reference DNA of the type strains of *Paenibacillus larvae* (DSM‐7030) and *Melissococcus plutonius* (DSM‐29964) was acquired from the German Collection of Microorganisms and Cell Cultures (DSMZ, Braunschweig, Germany). The PCR products were visualized by electrophoresis, and products showing single bands of the expected size were purified by ethanol precipitation and Sanger‐sequenced (Eurofins Genomics, Konstanz, Germany) to confirm their identity.

**Table 1:** Primers used in this study

Target	Forward primer 5′−3′	Reverse primer 5′−3′	Reference
Viruses			
Acute paralysis virus	TCATACCTGCCGATCAAG	CTGAATAATACTGTGCGTATC	Locke, Forsgren, Fries, and de Miranda (2012)
Black queen cell virus	AGTGGCGGAGATGTATGC	GGAGGTGAAGTGGCTATATC	Locke et al. (2012)
Chronic paralysis virus	CAACCTGCCTCAACACAG	AATCTGGCAAGGTTGACTGG	Locke et al. (2012)
Deformed wing virus A	TTCATTAAAGCCACCTGGAACATC	TTTCCTCATTAACTGTGTCGTTGA	Locke et al. (2012)
Deformed wing virus B	GCCCTGTTCAAGAACATG	CTTTTCTAATTCAACTTCACC	Locke et al. (2012)
Invertebrate iridescent virus 6	TGGTTYACCCAAGTACCKGTTAG	ATGCKGACCATTCGCTTC	Papp, Spann, and Marschang (2014)
Israeli acute paralysis virus	CCATGCCTGGCGATTCAC	CTGAATAATACTGTGCGTATC	Locke et al. (2012)
Kashmir bee virus	CCATACCTGCTGATAACC	CTGAATAATACTGTGCGTATC	Locke et al. (2012)
Lake Sinai Virus	TCATCCCAAGAGAACCAC	GCATGGAAGAGAGTAGGTA	This study
Sacbrood virus	TTGGAACTACGCATTCTCTG	GCTCTAACCTCGCATCAAC	Locke et al. (2012)
Slow paralysis virus	GCGCTTTAGTTCAATTGCC	ATTATAGGACGTGAAAATATAC	Locke et al. (2012)
Varroa destructor macula‐like virus	ATCCCTTTTCAGTTCGCT	AGAAGAGACTTCAAGGAC	Locke et al. (2012)
Bacteria			
Frischella perrara	GAAGCGAAGGTGCGAGCTGG	GTGGTAAACGCCCCCCTTGC	This study
Melissococcus plutonius	TGTTGTTAGAGAAGAATAGGGGAA	CGTGGCTTTCTGGTTAGA	Budge et al. (2010)
Paenibacillus larvae	CGGGAGACGCCAGGTTAG	TTCTTCCTTGGCAACAGAGC	Martínez, Simon, Gonzalez, and Conget (2010)
Parasites			
Crithidia mellificae, Lotmaria passim	CCGCTTTTGGTCGGTGGAGTGAT	GCAGGGACGTAATCGGCACAGTTT	This study, adapted from Meeus, De Graaf, Jans, and Smagghe (2010)
Nosema apis	CAGTTATGGGAAGTAACATAGTTG	CGATTTGCCCTCCAATTAATCTG	This study
Nosema ceranae	TGAGGCAGTTATGGGAAGTAATATTATATTG	ACTTGATTTGCCCTCCAATTAATCAC	This study
Acarapis woodii	GGAATATGATCTGGTTTAGTTGGTC	GAATCAATTTCCAAACCCACCAATC	Cepero et al. (2015)
Control genes			
Actin	TGCCAACACTGTCCTTTCTG	AGAATTGACCCACCAATCCA	Lourenço, Mackert, dos Santos Cristino and Simões (2008)
Elongation factor 1	GGAGATGCTGCCATCGTTAT	CAGCAGCGTCCTTGAAAGTT	Lourenço et al. (2008)
Ribosomal protein S5	AATTATTTGGTCGCTGGAATTG	TAACGTCCAGCAGAATGTGGTA	Evans (2006)
TBP‐association factor	TTGGTTTCATTAGCTGCACAA	ACTGCGGGAGTCAAATCTTC	Lourenço et al. (2008)

### 2.5 qPCR analyses

qPCR analyses were conducted on a Biomark HD system (Fluidigm, San Francisco, CA), strictly following the manufacturer's protocols for gene expression analysis. The quantitative performance of the essays were evaluated on FlexSix GE integrated fluidic circuits (IFC), where 12 replicates of the respective assay were matched with a 12‐step 10‐fold dilution series of the specific product with known sequence, mass, and concentration, to determine detection limits, linear dynamic detection range, variation at detection limit and PCR efficiency (see example evaluation data; Appendix 1). All assays showed PCR efficiencies above 90%, usually above 98%, and were able to detect 1–70 standard target molecules in the reactions, which, based on the molecular weight of the sequenced amplicons, corresponds to 36–250 target molecules per 100 ng of total RNA. cDNA was prepared from 200 ng total RNA using the Fluidigm Reverse Transcription Master Mix, which contains a mixture of poly‐T and random oligonucleotides. Negative controls were included on every plate, which underwent the same process as the samples during and after cDNA synthesis. Specific target enrichment with the Fluidigm Preamp Master Mix was carried out for 10 cycles with the pooled primers that were used later in the qPCR reactions (Table 1), thereafter the residual primers were digested with Exonuclease I (New England Biotech, Ipswitch, MA), and the resulting preamplified, ExoI‐digested samples were diluted fivefold. The final qPCRs were performed on 96 × 96 IFCs for gene expression (Fluidigm, San Francisco, CA). The manufacturer's standard qPCR protocol for fast PCR and melting curves was used (thermal mixing: 40 min 70°C, 30 s 60°C; hot start: 1 min 95°C; 30 cycles 5 s 96°C, 20 s 60°C; melting curve: 60–95°C 1°C/s). Automated quality control of the generated qPCR data was performed with the software Fluidigm Real‐Time PCR analysis. Only Cq‐values derived from reactions that showed a logarithmic increase in fluorescence and a specific melting temperature of the product, which was known from the sequenced standards, were used in the analysis. The results of the automated quality control were checked and revised manually to ensure high data quality. The lowest possible Cq‐value was 2.5 and the highest Cq‐values of approved positive reactions were around 27, corresponding to low numbers (1–50) of template molecules in some of the assays.

### 2.6 Data evaluation and statistics

Logarithmic linear regression was used for conversion of Cq‐values to *n*/reaction, which was in turn converted to *n*/100 ng RNA by back‐calculation of the dilution and preamplification steps. For visualization and comparison with other studies, virus concentrations were converted from *n*/100 ng RNA to *n*/bee by considering the average RNA yield of 1,423 ng/µl × 50µl = 71,150 ng RNA/bee, thus the conversion factor from *n*/100 ng RNA to *n*/bee is 711.5. All regression coefficients of the calibration functions were above 0.98. Logarithmic (log10) transformations of the concentration data were used for further statistical analysis. Differences in seasonal abundances were analyzed by comparing equal numbers of summer (May–August) and winter (November–February) samples from the regularly sampled colonies using a Mann–Whitney U test. Pairwise co‐occurrence of the abundant pathogens was analyzed by calculating Spearman's rank correlation coefficients. The least abundant organisms and abundance values below 1,000 target molecules/100 ng RNA were excluded from the correlation and regression analysis to eliminate uninfected individuals with low pathogen concentrations, as suggested by (Amiri, Meixner, Nielsen, & Kryger, 2015). To elucidate more complex patterns of co‐occurrence involving more than two pathogens, a multiple regression analysis with the six most abundant organisms (cutoff: 30% prevalence) was performed. DWV‐A and DWV‐B (Varroa destructor virus 1) were combined for regression analysis. A Kolmogorov‐Smirnov test indicated non‐normal distribution of residuals, thus a quadratic regression model was applied to identify significant predictors of each pathogen. To avoid overfitting of the model, predictor variables were entered stepwise into the model, in order of their predictive power, if they increased *F* by at least 0.05, and excluded if they increased *F* by <0.1. Independence of observations was checked using the Durbin‐Watson statistic. The robustness of the statistical analyses was tested by repetition with regional subsets of the data. All statistic calculations were done in SPSS statistics 25 (IBM).

## 3 RESULTS

**Figure 1:**
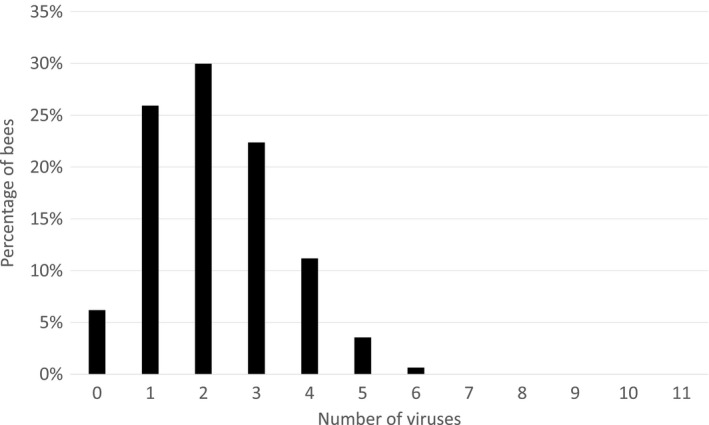
Numbers of different viruses detected per single bee (*n* = 1,064) throughout the study. Only 6% of the bees did not contain any virus, 68% of the bees contained two or more viruses, and 38% of the bees harbored three or more viruses. The average was 2.1 viruses/bee. The data distribution fits a Poisson model (*p* < .001), which indicates independent acquisition or infection events

Unexpectedly, every bee analyzed in this study was tested positive for at least one pathogen or parasite. Ninety‐four percent of the bees contained at least one virus, with black queen cell virus (BQCV) being the most prevalent (84%), followed by Lake Sinai Virus (LSV; 42%) and deformed wing virus type B (DWV‐B; 35%). In average, an individual bee carried 2.1 different viruses. Thirty‐eight percent of the bees contained three or more, 14% contained four to six different viruses (Figure 1). The prevalence and abundance of all assessed pathogens within the entire dataset are stated in Table 2.

**Table 2:** Prevalence and abundance of pathogens and parasites in this study

		*n* (bees analyzed)	*n* Positive	Prevalence	Mean	log (*n*/ 100 ng RNA)	
						Mean of positives	Maximum
Viruses	Black queen cell virus	1,064	895	84.1%	2.7	3.2	7.8
	Lake Sinai virus	1,064	446	41.9%	1.5	3.7	7.5
	Deformed wing virus B (VDV1)	1,064	376	35.3%	1.4	3.9	8.0
	Acute bee paralysis virus	1,064	173	16.3%	0.5	2.8	8.2*
	Chronic bee paralysis virus	1,064	164	15.4%	0.4	2.7	7.4
	Sacbrood virus	1,064	118	11.1%	0.2	2.2	6.7
	Deformed wing virus A	1,064	108	10.2%	0.4	3.9	7.2
	Aphid lethal paralysis virus	1,064	37	3.5%	0.0	1.3	3.6
	Israeli acute paralysis virus	1,064	14	1.3%	0.0	0.9	5.6
	Iridescent invertebrate virus IV	1,064	4	0.4%	0.0	1.0	1.2
	Kashmir bee virus	1,064	1	0.1%	0.0	1.3	1.3
Bacteria	*Frischella perrara*	1,064	824	77.4%	4.3	5.5	8.3*
	*Paenibacillus larvae*	1,064	295	27.7%	0.9	3.3	4.2
	*Melissococcus plutonius*	1,064	282	26.5%	0.8	3.1	4.3
Parasites	*Nosema ceranae*	1,064	1,026	96.4%	4.8	5.0	8.6*
	*Crithidia/Lotmaria*	1,064	553	52.0%	2.6	5.1	8.6*
	*Acarapis woodi*	1,064	158	14.8%	0.2	1.4	4.3
	*Nosema apis*	1,064	9	0.8%	0.0	2.6	6.3

* Maximum value of the analysis, corresponds to C_q_ = 2.5

The introduced intestinal parasite *N. ceranae* was ubiquitous (96%), while the actual endogenous species *N. apis* was only found in 1% of the bees, indicating that within the studied area *N. ceranae* has almost completely replaced *N. apis*. The trypanosomatid parasites *Crithidia mellificae* and/or *Lotmaria passim* were found in 52% of the bees. Since one assay was used for detection of both trypanosomatid species, they could not be distinguished in the analysis. Surprisingly, the tracheal mite *Acarapis woodi* was found in 15% of the bees, which is in contradiction to the commonly held belief that tracheal mites are now largely extinct. However, no seriously infested individuals were found, indicating either widespread resistance of the bees or effectiveness of the regularly used acaricides against tracheal mites.

**Figure 2:**
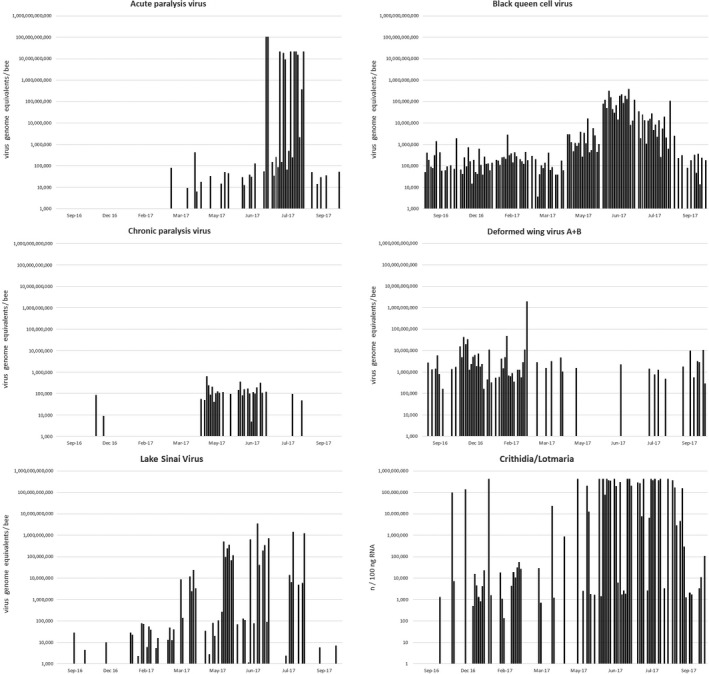
Seasonal dynamics of selected pathogens in one apiary. Each bar represents a single forager bee, and 18 bees from three colonies within the apiary were analyzed for each time point. Data from the same individuals are shown in all diagrams. The dynamics shown here are a good representation of the trends observed in the other apiaries assessed in this study, except for the paralysis viruses, which were absent or less abundant in most of the other apiaries

The American and European foulbrood bacteria, *Paenibacillus larvae* and *Melissococcus plutonius*, were present at low levels in about 27% of the samples; however, almost all of these foulbrood‐positive samples came from the same region in Western Baden‐Württemberg, while all but one of the other apiaries were completely free of foulbrood. Within this region, every apiary (14 out of 14) was tested positive for *Paenibacillus larvae* and *Melissococcus plutonius*. The two foulbrood bacteria were found to coexist at low levels in the same apiaries and colonies, but did rarely coincide in the same bees. Since they were detected by their ribosomal RNA, which is much more abundant on cellular level than DNA targets, the method is very sensitive and can detect very few cells per bee that may not be detected by other, DNA‐based methods. In one of the other apiaries acute and chronic paralysis virus infections arose during summer (Figure 2), however no symptomatic bees were noted during sampling, honey yield and colony strength were inconspicuous, and the sampled bees did not show any signs of disease. Apart from these regional differences in foulbrood bacteria and paralysis viruses, there were no striking differences in pathogen occurrences between apiaries.

The prevalent (>10%) viruses and parasites were analyzed for seasonal occurrence differences, and all viruses showed seasonal variation (Table 3, Figure 2). Acute and chronic paralysis virus, BQCV and LSV were more abundant during summer, while DWV and sacbrood virus (SBV) were more abundant during winter. Also the trypanosomatids *Crithidia/Lotmaria* and the tracheal mite *A. woodi* were more abundant during winter, while *N. ceranae* showed no pattern of seasonal variation. Seasonal differences in the foulbrood bacteria were not assessed, since the apiaries where they were predominantly detected were not sampled at all seasons. It should be noted that winter bees have a substantially extended lifespan, as compared to summer bees, therefore it is likely that more infections and higher pathogen concentrations can accumulate in winter bees.

**Table 3:** Seasonal differences in virus and parasite abundance

		ABPV	CBPV	DWV A+B	BQCV	LSV	SBV	Nosema ceranae	Crithidia/Lotmaria	Acarapis woodi
Number of cases	Summer	144	144	144	144	144	144	144	144	144
	Winter	144	144	144	144	144	144	144	144	144
Number of positive cases	Summer	61	35	29	141	67	6	135	69	4
	Winter	6	13	98	108	50	38	133	102	20
mean abundance all (*n*/100 ng RNA)	Summer	3,777,501	43	1,722	72,861	700,563	88	64,008,257	12,643,253	0.5
	Winter	0.8	10	2,868,649	2,295	71,490	27	62,776,162	137,774,717	2.5
median of positive cases (*n*/100 ng RNA)	Summer	124	145	4,072	9,033	516,749	335	13,849	6,297	22
*p*‐value: Mann–Whitney U test	Winter	13	35	2,304	254	73	94	20,077	124,109,755	12
		<.001	<.001	<.001	<.001	<.001	.002	.903	<.001	<.001

Abbreviations: ABPV, Acute bee paralysis virus; BQCV, Black queen cell virus; CBPV, Chronic bee paralysis virus; DWV, Deformed wing virus; LSV, Lake Sinai virus; SBV, Sacbrood virus

Pairwise Spearman's correlations were calculated for the abundant pathogens and parasites found in this study (Table 4). The highest correlation value was found for acute and chronic paralysis virus (0.5); however, this correlation is mostly based on the co‐occurrence of the two viruses in one apiary during summer (Figure 2) and should therefore be interpreted with care. *Frischella perrara*, which is a core member of the honeybee microbiome that causes tissue damage in the pylorus region of the gut (Engel, Bartlett, & Moran, 2015), was found in 77% of the bees. Despite its deleterious effect, there were no significant correlations of *F. perrara* with any pathogen or parasite, except sacbrood virus. This indicates that *F. perrara* is probably not a severe risk factor for development of disease. Black queen cell virus showed significant positive correlation with all other common viruses. This may indicate synergistic interaction, meaning that BQCV, which is found in every colony and almost every bee, may either tend to increase in the presence of other viruses, or that it may facilitate infection with or proliferation of other viruses, or both. Also, the intestinal parasites *N. ceranae* and *Crithidia/Lotmaria* showed robust correlation with BQCV and with LSV. However, most of the correlation coefficients are rather low (<0.4), which is an indication against strong mutual dependencies or pronounced synergistic effects. Also, the effects of synergism may for some pathogen pairs be confounded with the effects of seasonal co‐occurrence.

**Table 4:** Spearman's correlations of pathogen abundances in single forager bees calculated from log‐transformed concentration values. Zero values and measured concentrations below 1,000 target molecules/100 ng RNA were excluded from the analysis. Correlation values based on less than *n* = 50 data pairs are not shown. Significant (*p < .05) and highly significant correlations (**p < .001) are printed bold. The robustness of the correlations was tested by repeating the calculations with regional sub‐selections of the data. Correlations that were significant in all sub‐selections of the dataset are marked with a gray shades

	ABPV	CBPV	DWV A+B	BQCV	LSV	*Frischella perrara*	*Paenibacillus larvae*	*Melissococcus plutonius*	*Nosema ceranae*
CBPV									
DWV A+B	−0.04								
BQCV	0.05	0.25*	0.13**						
LSV		−0.09	−0.12	0.20**					
*F. perrara*	0.04	−0.12	0.06	0.06	−0.14*				
*P. larvae*			0.00	0.19**	−0.20	0.06			
*M. plutonius*			0.05	−0.31**	−0.16	0.10	0.04		
*N. ceranae*	0.04	−0.16	−0.02	0.16**	0.05	0.08*	−0.04	−0.07	
*Crithidia/Lotmaria*	−0.07	0.05	0.14*	0.25**	0.19**	−0.02	0.04	−0.16	−0.07

Abbreviations: ABPV, Acute bee paralysis virus; BQCV, Black queen cell virus; CBPV, Chronic bee paralysis virus; DWV, Deformed wing virus; LSV, Lake Sinai virus; SBV, Sacbrood virus.

To be able to detect patterns of correlation between more than two pathogens, a multiple regression analysis was conducted (Table 5). The abundant pathogens showed highly significant correlation with BQCV, which is in line with the pairwise correlations (Table 4). Also in reverse, BQCV was significantly correlated with DWV, LSV, *N. ceranae* and *Crithidia/Lotmaria*. Within the regression model, DWV was identified as the most significant predictor of BQCV, which is surprising since it has an opposing seasonal dynamic (Figure 2) and the Spearman's correlation value of BQCV with DWV was lower than for other pathogens (Table 4). However, the correlation and multiple regression analysis were recalculated with different regional subsets of the data and produced essentially the same results.

**Table 5:** Results of the multiple curvilinear (quadratic) regression analysis for BQCV, DWV, LSV, *Nosema ceranae*, *Crithidia/Lotmaria,* and *Frischella perrara*. Predictor variable was entered stepwise into the model and removed if they did not significantly increase the predictive power of the model

Dependent variable	Included predictor variables	*R*	*R^2^*	*p*‐value (ANOVA)	Sign. BQCV	Sign. DWV	Sign. LSV	Sign. N.cer	Sign. Crith	Sign. F.per
BQCV	DWV, LSV	.537	.289	<.001		<.001	<.001	.229	.109	.093
DWV	BQCV, LSV, Ncer	.547	.300	<.001	<.001		.001	.045	.445	.407
LSV	BQCV, DWV, Ncer	.497	.247	<.001	.003	.002		.019	.057	.219
*N. ceranae*	BQCV	.310	.096	.007	.007	.207	.140		.217	.976
*Crithidia & Lotmaria*	BQCV	.293	.086	.011	.011	.955	.126	.464		.935
*F. perrara*	DWV	.235	.055	.042	.279	.042	.352	.889	.828	

Abbreviations: BQCV, Black queen cell virus; DWV, Deformed wing virus; LSV, Lake Sinai virus; Ncer, Nosema ceranae.

*p*‐Values are derived from the *F*‐statistic of an ANOVA evaluating the goodness of fit of the regression model.

Significance of the respective predictor variable within the specified regression model.

## 4 DISCUSSION

The pathogen prevalences found in this study on the level of individuals exceed those found in most previous studies in collective samples, for example, Chen et al. (2004); Genersch et al. (2010); and Nielsen, Nikolaisen, and Kryger (2008), although similarly high prevalences were reported in France (Tentcheva et al., 2004). This may be due to the high sensitivity of the method used, which allows detection of less than 100 target molecules per 100 ng RNA (around 10^3^ target molecules per bee), and indicates that low‐level infections in honey bees are far more common than previously assumed.

Infections, or mere presence of a pathogen, as detected in this study, are not equivalent to disease. Asymptomatic or covert infections seem to be very common in honey bees, for example, about 15% of the bees assessed in this study were infected with acute and/or chronic paralysis virus, some at relatively high concentrations; however, none of them showed visible signs of disease (impaired movements, trembling, above‐average loss of hair). This absence of disease symptoms in infected bees was noted already when the paralysis viruses were first described (Bailey, Gibbs, & Woods, 1963) and affirmed in many subsequent publications, for example, Gauthier et al. (2007); Hung, Shimanuki, and Knox (1996); Molineri et al. (2017); Nielsen et al. (2008); and Tentcheva et al. (2004). Gauthier et al. (2007) quantified viral loads in asymptomatic bees by qPCR and found high concentrations similar to and even exceeding those reported here. Although we cannot exclude that the infected bees would have developed disease symptoms later on, the sampled foragers were already in the last weeks of their lives and it seems likely that many of them would have lived to the end of their short lifespan without developing disease symptoms. One factor that may play a major role for this phenomenon is the localization of the virus particles in the bodies of the bees; injection of very few virus particles results in symptomatic disease, while much higher amounts are necessary for infection by feeding or spraying (Bailey et al., 1963). For some of the viruses there are no known disease symptoms in adult workers, for example, BQCV, SBV, and LSV, and it is largely unknown if and how these viruses affect adult worker bees at all. However, if these viruses interact with other, more severe pathogens, they cannot be neglected. Since the present finding of multiple simultaneous virus infections in the same individual bee is in line with a previous study from the United States (Chen et al., 2004), it can be assumed that multiple virus infections on individual level are normal. Thus, studying interactions among the different abundant viruses and other pathogens is highly relevant for understanding the development of diseases.

Correlations and multiple regression analysis indicated that the prevalent viral pathogens and intestinal parasites are significantly correlated with BQCV. Although the correlation and regression coefficients were rather low (≤ +0.25), which refutes strong synergism or causality, the results indicate that there is some level of synergistic interaction among pathogens. The interaction is probably not direct, since that would likely result in a stronger association and correlation, but may be indirect by involving modification of the host's defense mechanisms. Deformed wing virus, the pathogen that had the highest explanatory significance for BQCV in the regression analysis, is thought to suppress the host's immune response when it has reached a certain critical level (Nazzi et al., 2012) and may therefore indirectly favor the proliferation of BQCV and other viruses. Similar effects of other viruses were described previously (Carrillo‐Tripp et al., 2016). In beekeeping practice, it is generally accepted that most diseases, which are not directly linked with mites, occur only under adverse conditions, such as in weak colonies, after periods of cold weather and when forage is limited. This suggests that the general physiological constitution of the bees, which probably determines the performance of their defense mechanisms, likely has an influence on pathogen proliferation.

Black queen cell virus resides and may propagate in the bee gut (Chen, Pettis, Collins, & Feldlaufer, 2006); therefore, a synergistic interaction with the intestinal parasites *N. ceranae* and *Crithidia/Lotmaria*, as indicated by the robust correlations that were observed, seems likely. Unfortunately, not much is known about the localization and tissue tropism of LSV, which could help explain its association with the intestinal parasites. A recent study indicated that the presence of the intestinal parasite *N. ceranae* promotes the proliferation of DWV, especially under nutritional stress (Zheng, Gong, Huang, & Sohr, 2015). Here, we did not find indications for pronounced synergism between DWV and *N. ceranae*. Seasonal differences in *N. ceranae* levels with a maximum in summer were reported from a large migratory beekeeper in the United States (Runckel et al., 2011); however in the present study, the overall levels of *N. ceranae* were constant throughout the seasons. While *N. ceranae* was ubiquitous in the study population, the inherently native species *N. apis* was only present in 1% of the bees, and exclusively in mixed infections with *N. ceranae*. This is in line with the previous finding that *N. ceranae* is replacing *N. apis* (Goblirsch, 2018; Paxton, Julia, Seppo, & Ingemar, 2007); however, its large prevalence and high concentrations in healthy, well‐performing colonies argue against the suspicion that *N. ceranae,* by itself, could be more virulent than *N. apis* (Higes, Garcia‐Palencia, Martin‐Hernandez, & Aranzazu, 2007; Paxton et al., 2007). It should be pointed out that since all of our measurements were done on RNA level, the reported concentrations of the organisms with a DNA genome (i.e., all bacteria and parasites) are not directly comparable to those reported in other studies from measurements on DNA level and give an indication of metabolic activity rather than cell numbers. Nevertheless, this does not dispute the validity of the reported prevalences.

The foulbrood bacteria *P. larvae* and *M. plutonius* were not evenly distributed within the sampled population, but occurred almost only in the westernmost region that was sampled. In this region, all of the fourteen sampled apiaries were positive for both foulbrood bacteria, while all other apiaries were free of *P. larvae* and mostly free of *M. plutonius*. This indicates that behind the visible outbreaks, which occur occasionally in the region, there may be an underlying, regionally distributed pathogen population that probably poses an additional threat to the bee colonies in the affected regions. From an epidemiological perspective, this finding raises concerns about the common practice of relocating bee colonies between different regions. In Germany, health assessment of bee colonies is mandatory before relocation; however, asymptomatic foulbrood infections cannot be detected by visual health checks, which are current practice. If molecular detection was implemented in health assessments, relocation of foulbrood‐positive colonies could be prevented, and the incidence of foulbrood outbreaks and the associated economic losses could be reduced.

In this study, common associations between pathogens and parasites on single‐bee level were identified, which may provide indication of synergistic interactions at individual level that underlie pathogenesis. However, the present study is limited to observing pathogen interactions that do not lead to rapid mortality of the affected bees. If a certain combination of pathogens would quickly cause the death of the affected bees, it is likely that the respective pathogens would be negatively correlated or not correlated at all, since no bees harboring both pathogens would remain alive and functional so they could be sampled. Also, other ways of pathogen interactions, for example, at colony level, may be possible, which cannot be assessed with the methods used in the present study. Further research involving multifactorial challenge experiments at individual and colony level would be required to clarify the full extent of synergism between pathogens, parasites, and environmental conditions in pathogenesis.

Here we have developed a comprehensive method for health assessment in large numbers of honey bee samples, which operates at a reasonable cost (<20 €/sample) and can be specifically adapted to a wide range of research objectives by changing or extending the panel of assays included. This analytic method could provide further insights into the complex pathology of honey bee colonies and may have potential for application in diagnostic routine. Above that, the present approach of assessing a wide panel of known targets in many samples by high‐throughput qPCR to gain enough statistic power for elucidating concealed patterns of interrelation may prove useful for other systems and sample types, and represents a flexible and cost‐efficient alternative to metagenomic and metatranscriptomic sequencing.

## AUTHOR CONTRIBUTIONS

PD and MH involved in study design, data analysis, and manuscript; PD, VS, KG, and MK involved in field samplings and laboratory work.

## Data Availability

All qPCR results (Cq‐value table) have been submitted to Dryad and will be published upon acceptance of the manuscript. The data can be accessed by the accession https://doi.org/10.5061/dryad.q7s60q9.

## Appendix 1

Evaluation of the BQCV assay. All qPCR assays were evaluated using 10‐fold dilution series of the respective amplicon (*n* = 12 per dilution). For all assays, the limit of detection was below 1,500 molecules/100 ng RNA, linear dynamic ranges extended over 5–6 orders of magnitude, and PCR efficiencies were above 90% (most assays: *E* > 98%).
